# Dietary Quercetin Regulates Gut Microbiome Diversity and Abundance in *Apis cerana* (Hymenoptera Apidae)

**DOI:** 10.3390/insects16010020

**Published:** 2024-12-28

**Authors:** Haodong Wu, Conghui Ji, Ruisheng Wang, Lijiao Gao, Wenhua Luo, Jialin Liu

**Affiliations:** Institute of Economic Animal, Chongqing Academy of Animal Sciences, Chongqing 402460, China; wuhaodong177@163.com (H.W.); j1621042024@163.com (C.J.); 13436082757@163.com (R.W.); gaolijiao002004@163.com (L.G.); luowh1966@126.com (W.L.)

**Keywords:** phytochemical, Eastern honeybee, gut microbiome, absolute quantification sequencing, health

## Abstract

Honeybee gut microbiota plays a crucial role in maintaining their health and digestive function. Studies have confirmed that quercetin improves honeybee health by enhancing their pesticide tolerance and survival rates. This study aimed to examine the effects of quercetin on the bee gut microbiome by absolute quantification sequencing. We included 1800 bees from the experimental apiary and exposed them to 151.2, 75.6, and 37.8 mg/L of quercetin. Gut samples were collected on the 5th and 9th days, subjected to a polymerase chain reaction and 16S rRNA sequencing, and analyzed. After 5 days of quercetin treatment, the diversity of the honeybee gut microbiota was altered, and total bacterial copies and *Lactobacillus* abundance significantly decreased at high quercetin concentrations (151.2 and 75.6 mg/L). On day 9, the gut microbial community had recovered from the adverse effects, and *Gilliamella* abundance increased in response to 37.8 mg/L quercetin treatment. However, quercetin had no noticeable effects on survival rate, food consumption, and gut structure. Our study confirmed the effect of short-term quercetin intake on the gut microbiota of *A. cerana*, providing valuable insights into how phytochemicals alter the bee gut microbiome, and their repercussions on host physiology.

## Simple Summary

Bees frequently ingest high concentrations of quercetin throughout their lifespan, as quercetin exists in pollen, nectar, and propolis. Studies have reported that quercetin enhances pesticide tolerance and survival rates in honeybees, contributing to their health. However, it is unclear whether quercetin affects their health via changes in the gut microbiota. This study demonstrated that short-term quercetin intake significantly altered the gut microbiome of *Apis cerana* at 5 and 9 days; however, quercetin had no significant effects on survival rate, food consumption, and gut structure. These findings improve our understanding of the interactions between dietary phytochemicals and the bee gut microbiome.

## 1. Introduction

Honeybees are essential to global ecosystems, serving as efficient pollinators and key drivers of ecological diversity [1]. In recent years, honeybee populations have faced severe global declines owing to mismanagement, disease, habitat degradation, pesticide exposure, and fluctuating food availability [2,3,4,5]. Meanwhile, the demand for insect pollination has tripled, raising concerns about an impending “pollination crisis,” where declining bee populations could reduce crop yields [6]. This loss poses serious threats to biodiversity and agriculture, potentially leading to ecosystem imbalances and grain reduction [7]. This has raised substantial concerns worldwide, making honeybee health enhancement a critical issue in both ecological and agricultural research.

The honeybee gut microbiome is crucial in maintaining the bees’ health and digestive function [8]. Similar to many social animals, honeybees acquire their gut microbiota through direct contact during social interactions [9,10], facilitating the establishment of a stable symbiotic relationship between the microbiota and the host [11,12]. The mature gut microbiome, comprising 10^8^–10^9^ bacterial cells [13], is generally established 6–8 days after emergence as adults [14,15]. The gut microbiota of worker honeybees is dominated by nine bacterial species clusters [10], with five core groups (*Snodgrassella alvi*, *Gilliamella apicola*, *Lactobacillus firm-4*, *Lactobacillus firm-5*, and *Bifidobacterium asteroids*) [16,17] comprising >95% of the total gut bacteria in nearly all individuals [18]. Wu et al. [19] reported that *A. cerana* and *A. mellifera* guts shared six dominant gut bacterial genera: *Lactobacillus*, *Bifidobacterium*, *Snodgrassella*, *bartonella*, *Gilliamella*, and *Frischella*; however, *A. cerana* hosted more *Apibacter*, while *A. mellifera* was predominantly colonized by *Commensalibacter*. Gut microbiota abundance is also influenced by various factors, including diet, age, seasonal changes, division of labor, and geographic factors [20,21]. Gut bacteria substrates predominantly originate from the diet of the host [22], thereby making diet the major modulator of the composition and metabolic activity of the gut microbiota [23,24].

Quercetin, a flavonoid widely found in plants, is among the most abundant plant phytochemicals [25]. Bee-collected pollen has been reported to contain quercetin concentrations ranging from 24.0 to 529.8 μg/g, with quercetin accounting for 0.0024–0.05% of pollen weight [26]. Since quercetin is not only enriched in pollen (beebread as a result) but also in nectar and propolis, honeybees are frequently exposed to high quercetin concentrations throughout their lifespan [27,28]. Johnson et al. [29] highlighted the protective role of quercetin in honeybees, particularly in mitigating tau-fluvalinate and imidacloprid toxicity. This effect is attributed to cytochrome P (CYP) 450 gene upregulation by phytochemicals, which encode the enzymes essential for detoxifying xenobiotics [30,31]. Another study demonstrated that phytochemicals enhanced honeybee survival rates regardless of pesticide exposure [32]. These findings demonstrate the importance of natural diets containing phytochemicals in pollinator health. *p*-Coumaric acid, another phytochemical in pollen and nectar, reportedly promotes the growth of microbes and improves honeybee health [33]. However, the effects of quercetin on the gut microbiome of pollinators remain poorly understood.

*Apis cerana* Fabricius (Hymenoptera: Apidae) is the predominant native pollinator of natural plants and agricultural cropping systems in Asia [34]. Millions of *A. cerana* colonies support pollination services for wild Chinese plants and crops [35,36]. Previous studies have confirmed that quercetin enhances pesticide tolerance of *A. cerana*. However, whether quercetin influences bee health through interactions with the gut microbiota remains underexplored. This study aimed to examine the impact of quercetin on gut microbiome diversity and abundance in *A. cerana* by absolute quantification sequencing; furthermore, we measured the potential impacts of quercetin on honey bee mortality, food consumption, and gut structure. By exploring the interactions between dietary phytochemicals and the gut microbiome of *A. cerana*, our findings offer insights into the potential factors that could improve honeybee health and survival.

## 2. Materials and Methods

### 2.1. Honeybees and Experimental Design

This study included three robust colonies maintained at the experimental apiary of the Institute of Economic Animals, Chongqing Academy of Animal Sciences, at Rongchang District, Chongqing, China. Each colony was equipped with a mated sister queen from 2024 and five bee frames (two brood and two honey and pollen frames). The apiary was located in a non-cultivated area, where colonies had no chance of chemical exposure before and during the experiments. To obtain adult workers of uniform age, sealed brood (pupal stage) frames were incubated overnight in a dark incubator at 34 °C and 70% humidity, as described by Liu et al. [37]. Newly emerged worker bees were marked with paint and returned to their colonies [38]. A 7-day period within the hive was sufficient for newly emerged bees to establish a natural gut microbiota level [15]; thus, marked bees were recaptured on day 7. The bees were then randomly assigned to single-use clear plastic rearing cages (Figure S1), each measuring 11 cm in diameter and 7 cm in height, featuring multiple ventilation holes and a larger opening for the insertion of a modified 10 mL syringe filled with 50% sucrose solution (*w*/*w*), ensuring 40 bees per cage. Before the formal experimental procedures, the bees were acclimated for 24 h by being fed sterile 50% sucrose solution (*w*/*w*) [39]. The control and treatment groups were conducted in parallel with five replicates, with 40 bees per replicate. A total of 1800 bees were included in this study.

### 2.2. Quercetin Effect on Worker Survival and Sucrose Solution Consumption

Three concentrations of quercetin (Sigma–Aldrich Co., Saint Louis, MO, USA, purity 95%) at 151.2, 75.6, and 37.8 mg/L, and a control group (CK), were used. Our previous studies demonstrated that these concentrations of quercetin effectively regulate pesticide tolerance in *A. cerana* [37]. The preparation procedures for treatment solutions containing phytochemicals have been previously described by Wong et al. [31]. A sterile sucrose solution was used to prepare the treatment solutions, each containing the corresponding phytochemical test concentrations and 0.25% dimethyl sulfoxide (DMSO) (*w/w*). Following a 24 h acclimatization, the bees were starved for 2 h, and then 4 mL of treatment solution containing DMSO with or without phytochemicals was randomly provided to each cage. The phytochemical treatments continued for 5 or 9 days. During the experimental period, the cages were maintained at 30 °C and 60% humidity. The treatment solution was refreshed and weighed daily, and dead bees were removed after daily survival checks.

### 2.3. Sampling and Tissue Sections

After the marked workers were recaptured from the colony, whole gut samples were immediately collected from 25 worker bees to establish a baseline control group, designated as 0 d. On the 5th and 9th days of quercetin treatment, whole gut samples were similarly collected from 25 workers in each treatment group. The dissection procedure involved cleaning and disinfecting the surface of the bees with 75% cold ethanol, drying using lint-free laboratory wipes, and carefully dissecting the whole gut under aseptic conditions [10]. A gut sample subset (each treatment condition of five replicates and five guts per replicate) was immediately frozen in liquid nitrogen after collection and stored at −80 °C for subsequent analyses. An additional 25 gut samples per treatment group were collected using the same method and fixed in 4% paraformaldehyde (Biosharp, Hefei, China) for tissue sectioning and hematoxylin–eosin (HE) staining, according to the protocol described by Libardoni et al. [40]. Images were captured using an Olympus BX53 inverted light microscope (magnification, ×200; Olympus Corporation, Tokyo, Japan).

### 2.4. DNA Extraction and Polymerase Chain Reaction (PCR) Amplification

Total microbial genomic DNA was extracted using the E.Z.N.A.^®^ soil DNA Kit (Omega Bio-tek, Norcross, GA, USA), according to the manufacturer’s instructions. Twelve spike-in sequences at four different concentrations (10^3^, 10^4^, 10^5^, and 10^6^ copies of the internal standards) were added to the sample DNA pools. These spike-in sequences included conserved regions matching selected natural 16S rRNA genes, while their artificial variable regions exhibited minimal sequence similarity with publicly available databases. These internal standards enabled absolute quantification of microbial DNA across samples. The hypervariable region V3–V4 of the bacterial 16S rRNA gene was amplified with primer pairs 338F (5’-ACTCCTACGGGAGGCAGCAG-3’) and 806R (5’-GGACTACHVGGGTWTCTAAT-3’) [41] by T100 Thermal Cycler PCR thermocycler (BIO-RAD, USA). The PCR reaction mixture included 4 μL 5 × Fast Pfu buffer, 2 μL of 2.5 mM deoxynucleotide triphosphates, 0.8 μL of each primer (5 μM), 0.4 μL Fast Pfu polymerase, 10 ng of template DNA, and double-distilled H_2_O to obtain a final volume of 20 µL. The PCR amplification cycling conditions were as follows: an initial denaturation at 95 °C for 3 min, followed by 27 cycles of denaturation at 95 °C for 30 s, annealing at 55 °C for 30 s, an extension at 72 °C for 45 s, a final single extension at 72 °C for 10 min, and ending at 4 °C. The PCR product was purified using a PCR Clean-Up Kit (YuHua, Shanghai, China), according to the manufacturer’s instructions, and quantified using Qubit 4.0 (Thermo Fisher Scientific, Waltham, MA, USA).

### 2.5. 16S rRNA Sequencing

Purified amplicons were pooled in equimolar amounts and sequenced using paired-end reads on an Illumina Nextseq2000 platform (Illumina, San Diego, CA, USA) according to the standard protocols of Majorbio Bio-Pharm Technology Co. Ltd. (Shanghai, China). Raw sequencing reads were deposited in the Sequence Read Archive database of the National Center for Biotechnology Information (Accession Number: PRJNA1183464).

After demultiplexing, the resulting sequences were quality-filtered using fastp (0.19.6) [42] and merged using FLASH (v1.2.11) [43]. High-quality sequences were denoised using the DADA2 [44] plugin in the Qiime2 (version 2020.2) [45] pipeline with the recommended parameters to obtain amplicon sequence variants (ASVs) with single-nucleotide resolution based on error profiles within samples. ASVs assigned to spike-in sequences were filtered out, and the reads were counted. A standard curve (Table S1), based on read counts versus spike-in DNA copy number, was generated for each sample, and the quantitative ASV abundance in each sample was determined. Taxonomic assignment of ASVs was performed using the naive Bayes classifier in Qiime2 and the SILVA 16S rRNA database (v138), with further adjustment for the evaluated rRNA operon copy number based on the *rrn*DB database [46].

### 2.6. Statistical Analysis

Statistical analyses were conducted using the SPSS 22.0 software (IBM, Armonk, NY, USA) and GraphPad Prism 8.0 software (GraphPad, San Diego, CA, USA). Kaplan‒Meier estimates were used to evaluate the survival rate differences of bees assigned to different feeding treatments. The treatment factor’s influence on *A. cerana* worker survival was analyzed using the log-rank test with Bonferroni correction. Sucrose solution consumption was compared using two-way repeated-measures ANOVA. The daily consumption values met normality, homogeneity, and sphericity assumptions.

Since the spike-in sequence was not derived from the sample, it was removed before subsequent analyses. Based on the ASV information, alpha diversity indices, including Shannon index, Simpson index, and Chao1 richness, were calculated using Mothur v1.30.1 [47]. Similarities among the microbial communities in different samples were determined via principal coordinate analysis (PCoA) based on Bray‒Curtis dissimilarity using the Vegan R-3.3.1 package. The permutational multivariate analysis of variance (PERMANOVA) was used to evaluate structural differences in the gut microbiota. Bacterial abundance data were considered non-parametric owing to a lack of normality. Kruskal‒Wallis ANOVA tests were used for comparison. The alpha level for all analyses was set to 0.05.

## 3. Results

### 3.1. Quercetin Did Not Affect Worker Survival and Sucrose Solution Consumption

**Figure 1:**
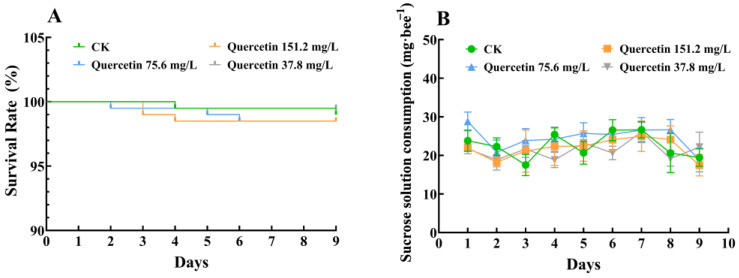
Worker survival rate and sucrose solution consumption. (**A**) Quercetin’s effect on honeybee survival is shown in Kaplan–Meier survival curves. (**B**) Sucrose solution consumption of honeybees under quercetin treatment over a 9-day period. Error bars showed the standard error of the mean. CK, control group. N = 5; 40 honeybees/replicate.

Worker bees were fed quercetin solutions at concentrations of 151.2, 75.6, 37.8, and 0 mg/L (control group) for 5 or 9 days. No statistical differences were observed in survival rate (Figure 1A; log-rank test: *χ^2^* =1.23; df = 3, 796; *p* = 0.75 > 0.05) and solution consumption (Figure 1B; two-way repeated measures ANOVA: F = 0.56; df = 3, 16; *p* = 0.95 > 0.05).

### 3.2. Quercetin Altered Gut Microbiota Abundance

**Figure 2:**
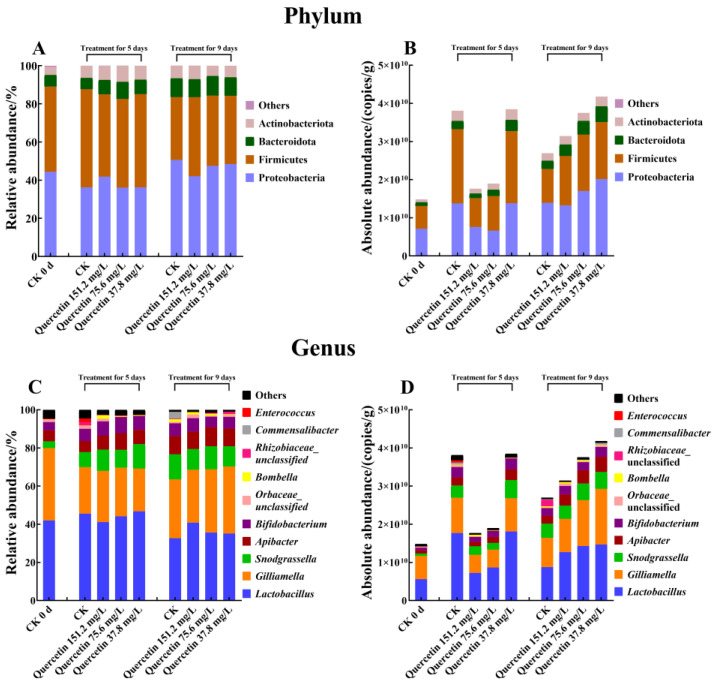
Classification analysis of the gut microbiota community of the worker bees at phylum and genus levels. (**A**) Relative abundance of dominant bacterial communities at the phylum level; (**B**) absolute abundance of dominant bacterial communities at the phylum level; (**C**) relative abundance of dominant bacterial communities at the genus level; (**D**) absolute abundance of dominant bacterial communities at the genus level. CK, control group.

Worker bee microbiota community structure was analyzed at both the phylum and genus levels. Regarding relative abundance, the results showed that the gut microbiota composition was similar across all bee groups (Figure 2A,C). At the phylum level, the dominant phyla were Proteobacteria (42.60%), Firmicutes (42.40%), Bacteroidota (8.29%), Actinobacteriota (6.58%), and other phyla (0.14%) (Table S2). At the genus level, the five most abundant bacteria were *Lactobacillus* (40.42%), *Gilliamella* (29.34%), *Snodgrassella* (10.08%), *Apibacter* (8.22%), and *Bifidobacterium* (6.57%) (Table S3). Regarding absolute abundance (Figure 2B,D), in the control group at 5 d (1.48 × 10^10^ ± 5.78 × 10^9^ copies/g), the total bacteria copies were significantly higher than that at 0 d (3.81 × 10^10^ ± 1.36 × 10^10^ copies/g) (*p* = 0.02 < 0.05); however, no significant difference was observed between that at 9 d (2.69 × 10^10^ ± 1.49 × 10^10^ copies/g, *p* = 0.54 > 0.05) and at 0 d (Kruskal‒Wallis: H = 7.22, df = 2, 12, *p* = 0.03 < 0.05).

**Figure 3:**
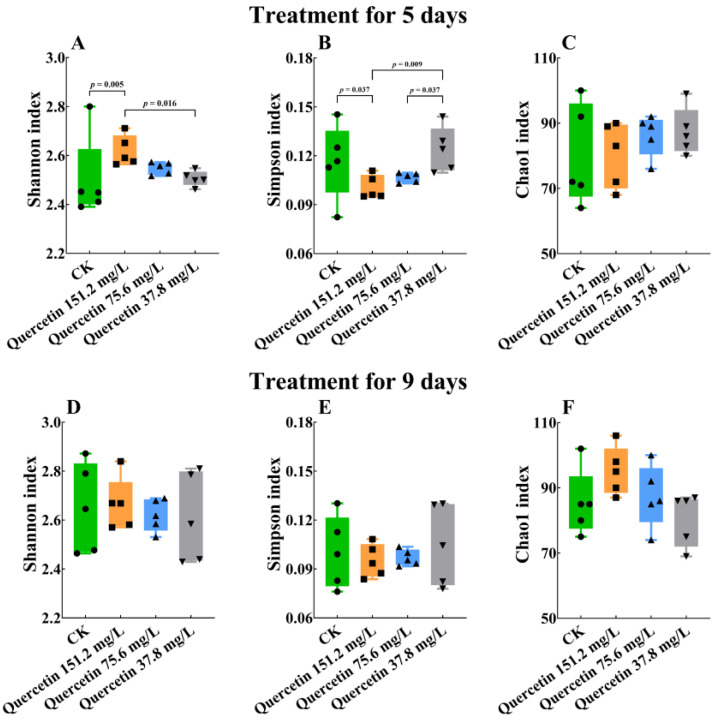
Alpha diversity between the control and quercetin treatment groups, measured by the Shannon, Simpson, and Chao1 indices. (**A**) Shannon index at 5 days. (**B**) Simpson index at 5 days. (**C**) Chao1 index at 5 days. (**D**) Shannon index at 9 days. (**E**) Simpson index at 9 days. (**F**) Chao1 index at 9 days. Tested for differences between groups using Kruskal‒Wallis analysis, α = 0.05. CK, control group.

Alpha diversity analysis revealed differences in the Shannon (Figure 3A; Kruskal‒Wallis: H = 9.65, df = 3, 16, *p* = 0.02 < 0.05) and Simpson (Figure 3B; Kruskal‒Wallis: H = 9.26, df = 3, 16, *p* = 0.03 < 0.05) indices after 5 days of quercetin treatment. The Shannon index for the 151.2 mg/L quercetin treatment group was prominently higher than that for the control (*p* = 0.01 < 0.05) and the 37.8 mg/L quercetin treatment groups (*p* = 0.02 < 0.05); however, no difference was observed with that of the 75.6 mg/L quercetin group (*p* = 0.26 > 0.05). The Simpson index in the 151.2 mg/L quercetin group was much lower than that in the control (*p* = 0.04 < 0.05) and the 37.8 mg/L quercetin (*p* = 0.01 < 0.05) groups. Moreover, the 75.6 mg/L quercetin group had a lower Simpson index than the 37.8 mg/L quercetin group did (*p* = 0.04 < 0.05); however, no difference was observed with that of the control group (*p* = 0.12 > 0.05). The Chao1 index showed no statistical difference between the different treatment groups on day 5 (Figure 3C; Kruskal‒Wallis: H = 1.43 df = 3, 16, *p* = 0.70 > 0.05). On day 9, no statistical difference was observed between the Shannon (Figure 3D; Kruskal‒Wallis: H = 0.20 df = 3, 16, *p* = 0.98> 0.05), Simpson (Figure 3E; Kruskal‒Wallis: H = 6.28, df = 3, 16, *p* = 0.10 < 0.05), and Chao1 (Figure 3F; Kruskal‒Wallis: H = 6.279, df = 3, 16, *p* = 0.010 < 0.05) indices.

**Figure 4:**
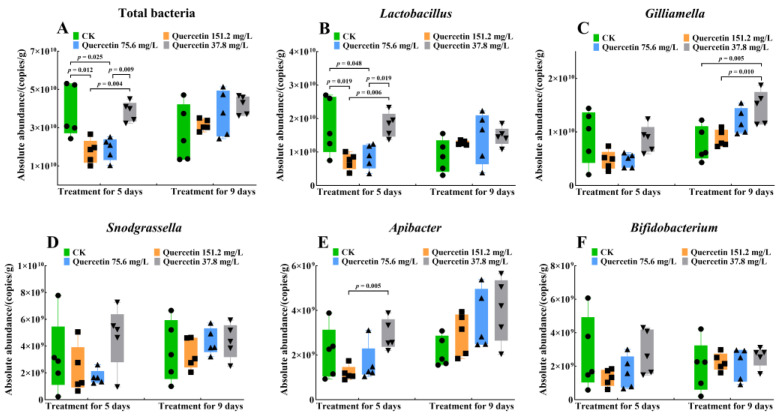
The absolute abundance of total bacterial copies and the five most abundant bacterial genera of *A. cerana* workers were measured as copies of the 16S rRNA gene. (**A**) Total bacteria copies; (**B**) *Lactobacillus*; (**C**) *Gilliamella*; (**D**) *Snodgrassella*; (**E**) *Apibacter*; (**F**) *Bifidobacterium*. Tested for differences between groups using Kruskal‒Wallis analysis, α = 0.05. CK, control group.

Subsequently, the total bacteria copies of the gut microbiota and the intergroup abundance differences of the five most abundant bacterial genera (*Lactobacillus*, *Gilliamella*, *Snodgrassella*, *Apibacter*, and *Bifidobacterium*) were examined. A discernible alteration in the total bacteria copies of the gut microbiota was observed after 5 days of quercetin treatment (Figure 4A; Kruskal‒Wallis: H =13.38, df = 3, 16, *p* = 0.01 < 0.05). Specifically, the control group exhibited significantly higher total bacterial copies than the 151.2 mg/L (*p* = 0.01 < 0.05) and 75.6 mg/L (*p* = 0.03 < 0.05) quercetin groups; however, no significant difference was observed between the control and the 37.8 mg/L quercetin groups (*p* = 0.71 > 0.05). *Lactobacillus* copies followed a similar trend of the total bacterial copies of gut microbiota (Figure 4B; Kruskal‒Wallis: H = 11.34, df = 3, 16, *p* = 0.01 < 0.05), wherein the control group showed significantly higher copies than the 151.2 mg/L (*p* = 0.02 < 0.05) and 75.6 mg/L (*p* = 0.048 < 0.05) quercetin groups. However, no significant difference was found between the control and the 37.8 mg/L quercetin (*p* = 0.71 > 0.05) groups. Furthermore, *Apibacter* showed an intergroup difference (Figure 4E; Kruskal‒Wallis: H = 8.26, df = 3, 16, *p* = 0.04 < 0.05). *Apibacter* abundance in the 37.8 mg/L quercetin group was higher than that in the 151.2 mg/L quercetin group (*p* < 0.01), whereas the difference between the control (*p* = 0.15 > 0.05) and the 75.6 mg/L (*p* = 0.07 > 0.05) quercetin groups was not significant.

On day 9, the results showed that only *Gilliamella* abundance differed significantly (Figure 4C; Kruskal‒Wallis: H = 10.58, df = 3, 16, *p* = 0.01 < 0.05). *Gilliamella* abundance in the 37.8 mg/L quercetin group was significantly higher than that in the control (*p* < 0.01) and the 151.2 mg/L (*p* = 0.01 < 0.05) quercetin groups. However, no intergroup differences were observed in *Snodgrassella* (Figure 4D; Kruskal‒Wallis: 5 d: *H* = 4.13, df = 3, 16, *p* = 0.25 > 0.05; 9 d: H = 1.70, df = 3, 16, *p* = 0.64 > 0.05) and *Bifidobacterium* (Figure 4F; Kruskal‒Wallis: 5 d: H = 3.11 df = 3, 16, *p* = 0.37 > 0.05; 9 d: H = 1.61, df = 3, 16, *p* = 0.66 > 0.05) abundance on either day 5 or 9.

However, in this study, quercetin did not significantly alter the relative abundance of dominant gut bacteria *Lactobacillus* (Kruskal‒Wallis: 5 d H = 3.46, df = 3, 16, *p* = 0.33 > 0.05; 9 d H = 3.80, df = 3, 16, *p* = 0.28 > 0.05), *Gilliamella* (Kruskal‒Wallis: 5 d H = 2.54, df = 3, 16, *p* = 0.47 > 0.05; 9 d H = 4.33, df = 3, 16, *p* = 0.23 > 0.05), *Snodgrassella* (Kruskal‒Wallis: 5 d H = 2.35, df = 3, 16, *p* = 0.50 > 0.05; 9 d H = 3.77, df = 3, 16, *p* = 0.29 > 0.05), *Apibacter* (Kruskal‒Wallis: 5 d H = 3.57, df = 3, 16, *p* = 0.31 > 0.05; 9 d H = 0.463, df = 3, 16, *p* = 0.93 > 0.05), and *Bifidobacterium* (Kruskal‒Wallis: 5 d H = 1.29, df = 3, 16, *p* = 0.73 > 0.05; 9 d H = 4.18, df = 3, 16, *p* = 0.24 > 0.05) in *A. cerana* (Figure S2).

**Figure 5:**
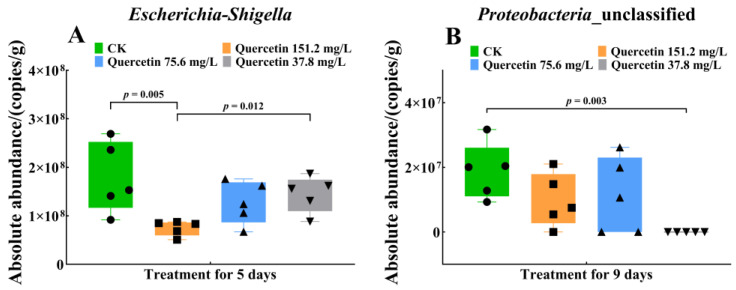
Absolute abundance of differentially non-dominant bacteria of *A. cerana* workers after quercetin treatment. (**A**) *Escherichia-shigella* copies differed after 5 days of quercetin treatment. (**B**) *Proteobactera*-unclassified copies differed after 9 days of quercetin treatment. Tested for differences between groups using Kruskal‒Wallis analysis, α = 0.05. CK, control group.

Among the non-dominant bacteria, differences in the abundance of one genus were observed between groups in the 5 d and 9 d quercetin treatment groups. On day 5, differences were observed in *Escherichia-Shigella* abundance (Figure 5A; Kruskal‒Wallis: H = 9.63, df = 3, 16, *p* = 0.02 < 0.05). *Escherichia-Shigella* absolute abundance in the 151.2 mg/L quercetin group was significantly lower than that in the control (*p* < 0.01) and the 37.8 mg/L (*p* = 0.01 < 0.05) quercetin groups. On day 9, differences were observed in *Proteobactera*-unclassified abundance (Figure 5B; Kruskal‒Wallis: H = 9.24, df = 3, 16, *p* = 0.03 < 0.05). The abundance in the 37.8 mg/L quercetin group was significantly lower than that in the control group (*p* < 0.01). However, no significant differences were observed between the control, the 75.6 mg/L (*p* = 0.07 > 0.05), and the 151.2 mg/L (*p* = 0.07 > 0.05) quercetin groups.

**Figure 6:**
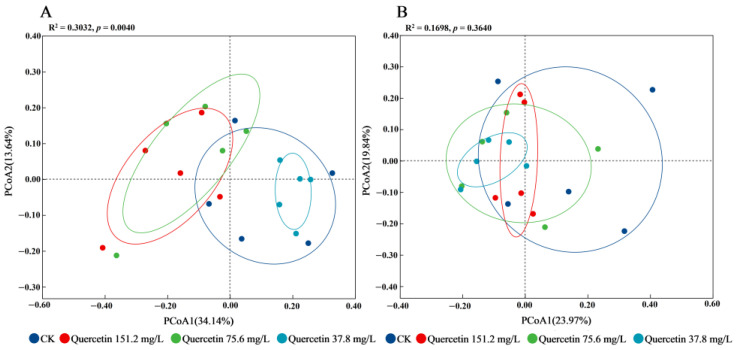
Beta diversity analysis using principal coordinate analysis (PCoA) based on the Bray‒Curtis dissimilarity index for absolute abundance at 5 days (**A**) and 9 days (**B**). Tested for differences between groups using PERMANOVA analysis, α= 0.05. CK, control group.

Beta diversity analysis using PCoA showed a significant separation in the community structure of gut microbiota among quercetin-treated samples on day 5 (Figure 6A; PERMANOVA: R^2^ = 0.30, F = 2.32, *p* = 0.004 < 0.05), indicating a notable alteration in species composition. However, no significant differences were observed at day 9 (Figure 6B; PERMANOVA: R^2^ = 0.17, F = 1.09, *p* = 0.36 > 0.05). These findings suggested that quercetin treatment induced significant changes in the gut microbiota composition on day 5, but this effect diminished by day 9.

### 3.3. Quercetin Did Not Affect Gut Structure

**Figure 7:**
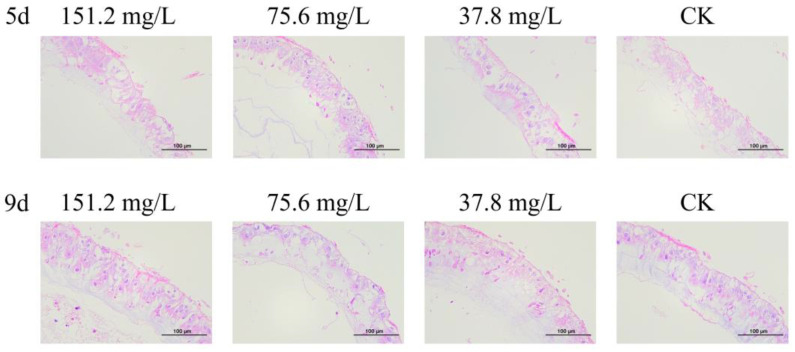
Cross sections of the gut of worker bees under quercetin treatment on days 5 and 9, with hematoxylin and eosin staining (200×).

In HE-stained sections, honeybee gut tissue exhibited a preserved architecture with a clear epithelial cell lining, intact columnar enterocytes, and nuclei. No cellular damage or necrosis was observed. After 5 or 9 days of quercetin treatment, intestinal morphology remained similar to that of the control, with intact structural integrity, cellular health, and no pathology signs (Figure 7).

## 4. Discussion

Phytochemicals extend honey bee lifespan and enhance their pesticide tolerance [32,48,49]. However, whether these compounds improve pollinator health via interactions with the gut microbiota remains underexplored. This study investigated the effects of quercetin on the diversity, abundance, and temporal dynamics of the gut microbiome of *A. cerana* by absolute quantification sequencing, alongside its impact on bee mortality, food consumption, and gut structure. Our findings confirmed that short-term phytochemical intake significantly impacted the gut microbiome of *A. cerana*. After 5 days of quercetin treatment, honeybee gut microbiota diversity altered, mainly because the high quercetin concentration (151.2 and 75.6 mg/L) treatment reduced the total bacterial copies and *Lactobacillus* abundance. On day 9, the microbial community had largely recovered, with an increase in *Gilliamella* observed in the 37.8 mg/L quercetin treatment group. However, no significant effects on workers’ survival, food consumption, or gut structure were observed.

The total bacterial copies and *Lactobacillus* abundance decreased significantly at high concentrations after 5 days of quercetin treatment. *Lactobacillus* accounted for the largest proportion of the gut microbiota composition, approximately 32.69–46.77%, highlighting the pivotal role of *Lactobacillus* in shaping the overall microbial landscape of honeybee guts. As observed in our study, the reduction in *Lactobacillus* copies also resulted in a decreased total number of gut bacteria. Various *Lactobacillus* species have been reported to provide specific health benefits to honeybees, induce immune stimulation (antimicrobial peptide upregulation) to enhance resistance to foreign pathogens [50,51,52], and reduce the toxicity of pesticides and antibiotics on honeybees [53]. *Lactobacillus*, which participates in the digestive process, possesses glycosidase activity that breaks down complex polysaccharides and metabolizes toxic sugars [54,55]. In addition, Zhang et al. [56] found that *Lactobacillus lactis* modulates host learning and memory behaviors by regulating tryptophan metabolism. Therefore, a short-term (5 days) quercetin-induced reduction in the *Lactobacillus* population could disrupt the symbiotic balance within the gut microbiome, affecting metabolic functions, immune response modulation, and cognitive abilities.

On day 9, the honeybee gut microbial community showed signs of recovery, characterized by a significant increase in *Gilliamella* abundance at a low quercetin concentration (37.8 mg/L). This result suggested that *Gilliamella*, a core symbiont, may play a crucial role in quercetin metabolism. *Gilliamella* is essential for carbohydrate metabolism and regulates the glycerophospholipid pathway [57]. Specifically, *Gilliamella apicola* assists in metabolizing carbohydrates that could otherwise be toxic to its host [57]. Owing to the absence of specific digestive enzymes, certain monosaccharides are indigestible to social bees, including arabinose, mannose, galactose, rhamnose, and xylose [58]. *Gilliamella* assists the host in degrading and utilizing these sugars, and metabolizing pectin [59,60]. In addition, *Gilliamella* has been linked to the bee’s response to dietary stress, such as high-fat diets, where its abundance increases as observed by Wang et al. [61]. These findings indicate that *Gilliamella* makes an important contribution to honeybee dietary tolerance and resilience.

Despite the perturbation caused by high quercetin concentrations over a short time frame (5 days), honeybees exhibited strong recuperative abilities in their gut microbiota composition. By day 9, the bees showed signs of alleviating the adverse effects of quercetin. We speculated that the detoxification enzymes and adaptive microbial shifts play essential roles in alleviating the adverse effects of quercetin. Studies have shown that honeybee CYP450 monooxygenases are involved in detoxifying pesticides and phytochemicals, including quercetin [62,63]. Specifically, enzymes from the CYP6 subfamily, such as CYP6AS1, CYP6AS3, CYP6AS4, and CYP6AS10, are crucial for efficient quercetin metabolism [27]. Furthermore, several studies have confirmed that the honeybee gut microbiota can metabolize xenobiotics, including various phytochemicals [63,64,65]. For example, Motta et al. [66] identified strains of *Bifidobacterium*, *Bombilactobacillus*, and *Gilliamella* that facilitated the degrading of amygdalin. The metabolism of phytochemicals by detoxifying enzymes and gut microbes likely mitigates the adverse impact of quercetin on the honeybee gut microbiome. However, since the detoxification pathway of quercetin in honeybees is not yet fully understood, further research is required to understand their adaptive mechanisms to quercetin.

Host microbiota modulate nutrition–immunity–pathogen relationships [67]. However, our study found no significant effect of gut microbiome alteration induced by short-term quercetin intake on survival rate, food consumption, and gut physiological structure in *A. cerana* workers. A similar short-term survival experiment was conducted by Liao et al. [32] on *A. mellifera* over 8 days, and quercetin increased the survival rate. This suggests that the adaptabilities of *A. cerana* and *A. mellifera* to phytochemicals may differ. However, we observed that the test bees in that experiment lacked gut microbiota colonization. Several studies investigating the catabolism of quercetin and its glycosides by fecal microbiota have highlighted the critical role of gut microbes in phytochemical metabolism [68,69,70,71]. This suggests that the presence or absence of gut microbiota colonization could be a contributing factor to the different observed outcomes. In future studies, the colonization of gut microorganisms should be considered when exploring the effects of phytochemicals on bees.

Absolute quantification sequencing provided a richer source of information in this study, although most previous microbiome studies focus on relative abundance measurements [72,73,74]. In our analysis, relative quantification failed to reveal significant differences, whereas measuring the total bacterial copies and abundance of gut microbes accurately reflected actual fluctuations. In absolute quantification, we observed that the total gut bacteria copies in workers was higher on day 5 than on day 0, suggesting that gut microbes continued to proliferate rapidly after the end of the 7-day hive colonization period. This further confirmed that gut microbiota copies vary with honeybees’ daily age [38,75], underscoring the necessity of consistent age selection in gut microbiota studies. Moreover, the honeybee gut microbial spectrum was conserved and stable, with dominant bacterial genus including *Lactobacillus*, *Snodgrassella*, *Frischella*, *Gilliamella*, and *Bifidobacterium*, alongside a few non-core members such as *Apibacter* [76,77]. The composition of the honeybee gut microbiota remained relatively stable across groups in this study, consistent with previous studies [78,79,80], supporting the stability and reliability of the reference colonization method [38].

This study had some limitations. Although we identified alterations in gut microbiota following short-term quercetin intake, no notable physiological effects on worker bee survival, food consumption, or the gut structure of *A. cerana* were observed. This suggests that quercetin requires a longer duration to exert its beneficial effects [75]. In addition, gut bacterial colonization may be an important factor that impacts the effect of quercetin on honeybee health. Moreover, the laboratory cage experiments in our study cannot replicate the complex conditions present in the natural environment. Future studies should extend the observation periods and integrate field experiments to examine the response patterns and adaptive strategies of bees toward quercetin in natural environments.

## 5. Conclusions

This study revealed that a short-term quercetin intake caused significant changes in the *A. cerana* gut microbiome. Five-day treatment with high quercetin concentrations (151.2 and 75.6 mg/L) resulted in a marked alteration in gut microbiome diversity and total bacterial load decline, particularly in *Lactobacillus* abundance. Intriguingly, on day 9, these alterations and reductions were recovered, and *Gilliamella* abundance significantly increased in the 37.8 mg/L quercetin treatment group. Despite shifts in the gut microbiota, no significant effects on survival, food intake, or gut morphology were observed. This suggests that the short-term effects of quercetin predominantly manifest as alterations in gut microbiota composition rather than as direct physiological changes. Collectively, our study highlighted the role of dietary phytochemicals in modulating pollinator gut microbiota and potentially influencing health via microbiome interactions.

## Data Availability

The original contributions presented in the study are included in the article/Supplementary Material. Further inquiries can be directed to the corresponding author.

## Supplementary Materials

The following supporting information can be downloaded at: https://www.mdpi.com/article/10.3390/insects16010020/s1, Table S1: The standard curve based on read counts versus spike-in DNA copy number of each sample. Table S2: Relative abundance of major bacterial phyla based on copy number analysis; Table S3: Relative abundance of major bacterial genera based on copy number analysis; Figure S1. Images depicting (**A**) the rearing cage used for honeybee experiments and (**B**) the placement of rearing cages in an incubator; Figure S2. The relative abundance of dominant bacterial genera of worker bees.
