# Supplementary material for: Dietary Quercetin Regulates Gut Microbiome Diversity and Abundance in Apis cerana (Hymenoptera Apidae)
Source: Insects. 2024 Dec 28;16(1):20. doi: 10.3390/insects16010020 (PMC11766270; doi:10.3390/insects16010020)
Supplement: Supplementary file 1 [file insects-16-00020-s001.zip › insects-3378151-supplementary.pdf]

Table S1. The standard curve based on read counts versus spike-in DNA copy number of each sample

Value	0d CK	5d CK	5d Q1	5d Q2	5d Q3	9d CK	9d Q1	9d Q2	9d Q3
**a**	0.9496	0.9582	0.9482	0.9499	0.9514	0.9573	0.9380	0.9399	0.9471
**b**	2.5580	2.5683	2.6066	2.5166	2.6436	2.5944	2.6338	2.6942	2.6731
R^2	0.9859	0.9895	0.9826	0.9842	0.9832	0.9860	0.9825	0.9803	0.9815

a, the slope of the standard curve; b, the intercept of the standard curve; R^2, the determination coefficient of the standard curve. CK, control group; Q1, 151.2 mg/L quercetin treatment group; Q2, 75.6 mg/L quercetin treatment group; Q3, 37.8 mg/L quercetin treatment group.

Table S2. Relative abundance of major bacterial phyla based on copy number analysis

Taxon	Proteobacteria	Firmicutes	Bacteroidota	Actinobacteriota	Others
**0d CK**	44.4046%	44.6527%	6.0114%	4.2896%	0.6417%
**5d CK**	36.2298%	51.4054%	5.8483%	6.4572%	0.0592%
**5d Q1**	41.8418%	43.1169%	7.4775%	7.4820%	0.0818%
**5d Q2**	36.0955%	46.4840%	8.9009%	8.4300%	0.0897%
**5d Q3**	36.2103%	48.8773%	7.5238%	7.2959%	0.0927%
**9d CK**	50.5776%	32.9719%	9.7045%	6.6713%	0.0746%
**9d Q1**	42.0388%	41.4493%	9.3686%	7.0800%	0.0632%
**9d Q2**	47.5074%	36.8144%	10.1998%	5.4148%	0.0636%
**9d Q3**	48.4601%	35.7636%	9.5840%	6.1017%	0.0905%
**Average**	42.5962%	42.3928%	8.2910%	6.5803%	0.1397%

CK, control group; Q1, 151.2 mg/L quercetin treatment group; Q2, 75.6 mg/L quercetin treatment group; Q3, 37.8 mg/L quercetin treatment group.

Table S3. Relative abundance of major bacterial genera based on copy number analysis

Taxon	*Lactobacillus*	*Gilliamella*	*Snodgrassella*	*Apibacter*	*Bifidobacterium*	*Orbaceae*_unclassified	*Bombella*	*Rhizobiaceae*_unclassified	*Commensalibacter*	*Enterococcus*	Others
**0d CK**	41.9848%	37.9329%	3.5151%	5.8407%	4.2810%	1.3190%	0.0411%	-	0.0073%	0.2968%	4.7813%
**5d CK**	45.5450%	24.4113%	7.7967%	5.8444%	6.4458%	1.3282%	0.3265%	1.7555%	-	1.8855%	4.6611%
**5d Q1**	41.1026%	26.9072%	11.0096%	7.4489%	7.4748%	1.2811%	1.8328%	0.1030%	0.0019%	0.0289%	2.8094%
**5d Q2**	44.1234%	25.5151%	9.3337%	8.8169%	8.4285%	0.0605%	0.4057%	0.0011%	0.0015%	0.2447%	3.0689%
**5d Q3**	46.7722%	22.4190%	12.7790%	7.5156%	7.2906%	0.0932%	0.2682%	0.0013%	0.0058%	0.0587%	2.7963%
**9d CK**	32.6871%	30.8119%	13.0794%	9.6785%	6.6702%	0.6102%	1.3640%	0.2958%	3.6025%	0.0044%	1.1960%
**9d Q1**	40.8194%	27.7397%	10.7523%	9.2236%	7.0747%	1.8262%	1.2260%	-	0.0598%	0.0420%	1.2362%
**9d Q2**	35.6220%	33.2037%	11.9656%	10.1917%	5.4044%	0.4555%	0.9699%	0.5715%	-	0.0178%	1.5980%
**9d Q3**	35.1467%	35.1459%	10.4630%	9.4512%	6.0967%	0.9343%	0.3490%	1.2771%	-	0.0121%	1.1240%
**Average**	40.4226%	29.3430%	10.0771%	8.2235%	6.5741%	0.8787%	0.7537%	0.4450%	0.4088%	0.2879%	2.5857%

- means no copies of this bacterial genus were measured in the gut samples. CK, control group; Q1, 151.2 mg/L quercetin treatment group; Q2, 75.6 mg/L quercetin treatment group; Q3, 37.8 mg/L quercetin treatment group.

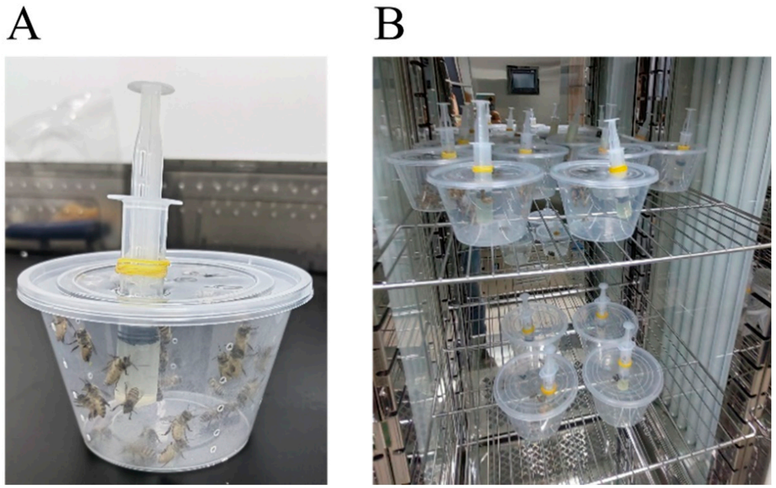

Figure S1. Images depicting (A) the rearing cage used for honeybee experiments and (B) the placement of rearing cages in an incubator

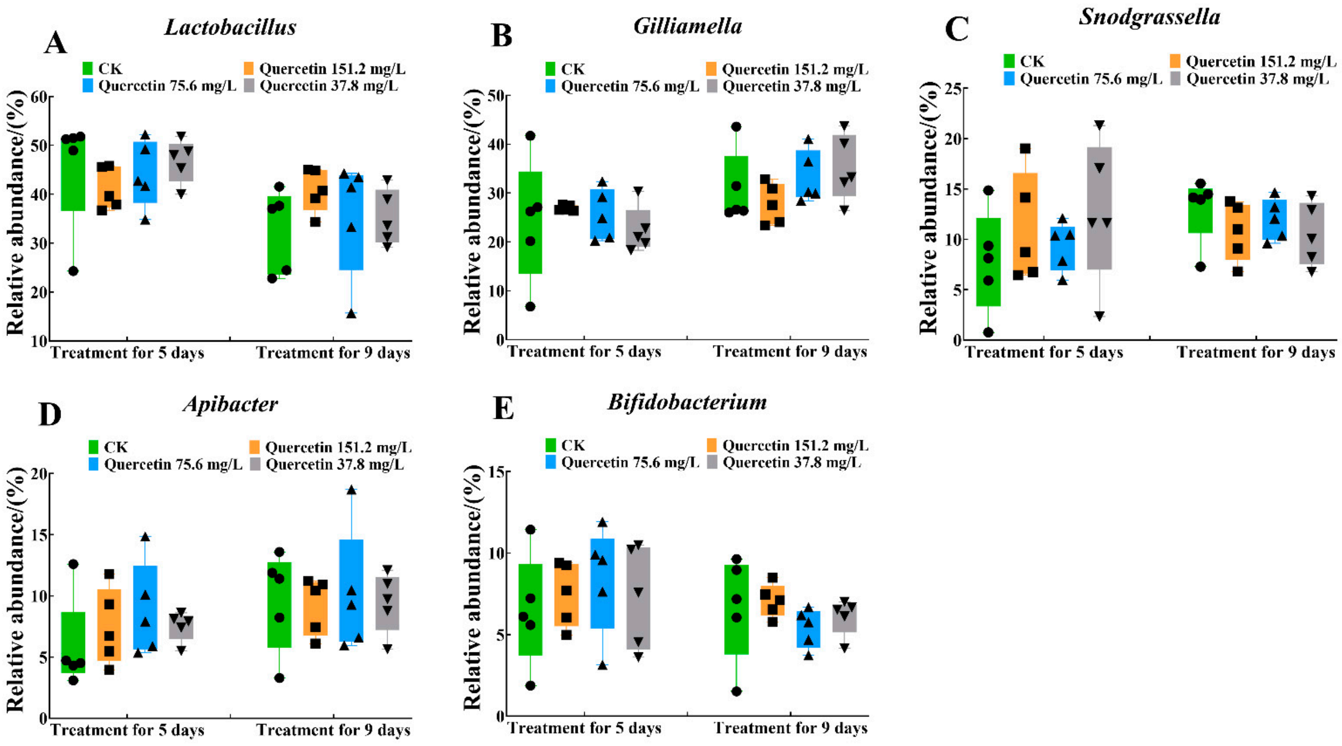

Figure S2. The relative abundance of dominant bacterial genera of worker bees.
